# Governance and human resources for health

**DOI:** 10.1186/1478-4491-9-29

**Published:** 2011-11-24

**Authors:** Marjolein Dieleman, Thea Hilhorst

**Affiliations:** 1 Royal Tropical Institute, Amsterdam, the Netherlands

## Abstract

Despite an increase in efforts to address shortage and performance of Human Resources for Health (HRH), HRH problems continue to hamper quality service delivery. We believe that the influence of governance is undervalued in addressing the HRH crisis, both globally and at country level. This thematic series has aimed to expand the evidence base on the role of governance in addressing the HRH crisis. The six articles comprising the series present a range of experiences. The articles report on governance in relation to developing a joint vision, building adherence and strengthening accountability, and on governance with respect to planning, implementation, and monitoring. Other governance issues warrant attention as well, such as corruption and transparency in decision-making in HRH policies and strategies. Acknowledging and dealing with governance should be part and parcel of HRH planning and implementation. To date, few experiences have been shared on improving governance for HRH policy making and implementation, and many questions remain unanswered. There is an urgent need to document experiences and for mutual learning.

## Editorial

Although efforts to address shortage and performance of Human Resources for Health (HRH) have accelerated over recent years, HRH problems continue to hamper the goal of quality service delivery [1]. Currently, fifty-seven countries face a critical workforce shortage and many more countries are not able to provide quality care to their population because of workforce problems [2]. Why is there little progress in addressing the HRH crisis?

Is governance the elephant in the room of HRH? Do we prefer not to mention it? Or do we have the different parts in our hands but are not able to assemble it to make it work for better results? We believe that the influence of governance is undervalued in the debate on the HRH crisis, both globally and at country level. This thematic series of the HRH journal aims to expand the evidence base on the role of improving governance in addressing the HRH crisis.

Six articles on governance in HRH have been brought together for this series: a review of published case studies on HRH and governance [3]; a case study on HRH policy formulation and implementation in post-conflict Liberia [4]; a commentary on opportunities for HRH policy in meeting population needs in a decentralized setting in Mali [5]; monitoring HRH and the use of a Human Resources Information Systems from a regional perspective [6]; a case study on Human Resources Management in a decentralized context in Brazil [7] and measuring contributions of development partners to financing of HRH activities [8].

These articles were presented in the conference "Responsible governance for improved human resources for health: making the right choices" organised by the Royal Tropical Institute in Amsterdam in 2010. In this conference, 181 people from 31 countries participated to discuss and exchange their experiences with governance-related issues for HRH. Five governance areas were distinguished: "development of a vision and policies for HRH"; "aid effectiveness"; "regulatory mechanisms"; "participation and voice" and "governance in competency development in higher education for public health". During the conference, the following definition of governance was used as the entry point because it puts actors, their roles and power at the center: "Governance is about the rules that distribute roles and responsibilities among government, providers and beneficiaries and that shape the interactions among them. Governance encompasses authority, power, and decision-making in the institutional arenas of civil society, politics, policy, and public administration" [9]. The conference demonstrated that many efforts have been undertaken at country level to analyse and bring to the fore governance related issues in HRH policy formulation and implementation, and to describe efforts to improve governance structures and strategies in HRH [10].

The first paper in this series is a review of published case studies on HRH and governance [3]. Few cases exist that address governance, a term used in many different ways. Most cases focus on vision and policies for HRH, and on aid-effectiveness and partnerships with development partners. Limited studies are available on stakeholder participation, users' or health workers' voice and agency, or on regulatory mechanisms. A crosscutting theme was governance challenges in relation to local level corruption, which in turn undermines accountability and mutual trust. Decision-making processes to select and develop HRH policies often are non transparent. More clarity on such processes would allow increased understanding on why certain policies are successful and others not. The review did not identify any case studies on decision-making processes for HRH. This gap could be filled by undertaking political economy analysis in the field of HRH, which analyses the influence the context, actors and processes have on each other in policy-making [11]. Such insights can assist policymakers and planners to better plan the process, to assess resistance and support and to estimate leeway in negotiations. As such it can also be a good starting point to assess feasibility of certain policy decisions and prepare grounds for developing strategies to meet resistance. It is important to acknowledge that power relations among stakeholders in HRH exist and that these influence decision-making in HRH. Dealing with resistance to change not only requires insight into power relations but also insight into opportunities to influence decisions on HRH policy formulation.

The second case study on Liberia presents a comprehensive overview of how HRH policies were developed and implemented in a post-conflict setting [4]. Here, the Ministry set up a well coordinated process for HRH policy formulation, involving a wide variety of actors. The article demonstrates also the importance of committed leadership, which was instrumental in building partnerships and arriving at a common vision. This then enabled the combining of resources to fund a clear HRH plan. A number of important HRH results were achieved, such as a 73% increase in availability of nurses, improved pre-service training and the establishment at national and county level of HRH structures with competent personnel.

Strengthening domestic accountability is even more important for responsive service delivery. One article looks at accountability for HRH at the local level: efforts to decentralize HRH functions in Mali are discussed in the commentary by Lodenstein and Dao [5]. The authors describe the opportunities that devolution (i.e. decentralization to local government) offers in terms of better meeting the needs of the local population, but explain that more attention needs to be paid to public accountability and innovative capacity development efforts, as these are crucial if change in quality and equity of staff is to be obtained.

For HRH plans to be evidence-based and addressing key bottlenecks, a functioning information system on service delivery is crucial. This needs to be complemented with research. Equally, HRH plans need to be informed by global evidence on what works under which circumstances. The limitations of current information systems for HRH have been highlighted [2] and were also a recurrent concern at the KIT HRH conference in 2010 and the Global HRH forum in Bangkok in 2011. The article of Nigenda et al. addresses information systems and indicators from a regional perspective [6]. The challenge is the selection of key indicators that provide management and policy makers with useful information for decision-making and for which regular data collection is feasible with the resources available. The article explains how nine countries in Latin America and the Caribbean developed together common metrics for HRH. Taking a regional level perspective facilitates comparisons and benchmarking, and capacity development. The starting point was an inventory of published HRH metrics, which showed that most information systems or studies only cover a part of the HRH area. Most systems focus on the labour market, followed by monitoring of working conditions and of training. The article of Nigenda et al. reiterates again the gap in knowledge on the HRH situation in various countries, and the importance of formulating a range of key indicators and a metrics for planning.

The organisational structure of the Ministry of Health needs to take into account HRH strategy development and planning of implementation. However, in many countries responsibilities related to HRH are distributed among different departments and even ministries, such as responsibilities for planning, personnel management, for pre-service training, or professional development. The article of Pierantoni and Garcia shows how limited management competencies can hamper the implementation of planned policies [7]. Despite the intentions to expand HR functions at decentralized levels from personnel administration to more comprehensive HRH management, insufficient investments were made to assure that HRH units had managers with the required competencies. Pierantoni and Garcia show further that both management competencies and governance structures are needed for effective policy formulation and implementation. This requires investment in capacity building and strengthening of governance structures. In addition, more effective and efficient HRH planning and management requires the establishment of HRH units located in such a way that there is influence on the health system, and which are equipped with competent personnel and committed and persevering leadership to assure appropriate resource allocation and the accommodation of resistance. However, a WHO study showed that HRH units often are located at a lower level in the health sector, implying limited influence and that these units often have a high turnover of directors, demonstrating instability [12].

Another component of effective HRH policies and plans is the management of external funds, as well as the implementation of the aid effectiveness agenda, as demonstrated in the article of Campbell et al [8]. In reviewing official development assistance to HRH through an analysis of UK government contributions, it turned out to be difficult to evaluate the results of donor engagement. Although DfID has invested more in HRH and aligned with WHO's recommended '50:50 principle' to assist in addressing the HRH crisis, it was not able to show an increase in actual spending and account for the results on HRH. The reason is that the types of indicators used to measure aid (OECD's Creditor Reporting System) are not specific enough for HRH, and a so-called "rational approach" for estimating HRH investment had to be used. This raises questions for the managing for results elements and the mutual accountability of the "Paris Declaration". If there are no reliable basic data on the national workforce, recurrent costs and domestic and external financing then accountability and transparency are hampered, and effectiveness cannot be assessed. Campbell et al. conclude that the lack of data on aid for HRH is a governance issue, and suggest that the G8 and other development partners could use the Agenda for Global Action, as a structured monitoring tool. In turn, such evidence could subsequently leverage "more money for HRH" from both domestic and international resources.

The six articles published in this thematic series present a range of experiences which take into account governance issues upfront in addressing the HRH crisis. The articles report partly on governance in relation to developing a joint vision, building adherence and strengthening accountability, and partly on governance with respect to planning, implementation, and monitoring. They cover elements related to two out of the five governance areas defined at the KIT HRH 2010 conference: "development of a vision and policies for HRH" and "aid effectiveness". Other governance issues warrant attention as well. For instance, a number of case studies described in the review article [3] highlighted that HRH policy formulation and implementation often lack transparency and suffer from corruption, and more insight into effective mechanisms and instruments for addressing these challenges is required. More attention also needs to be paid to documenting experiences covered by the areas "regulatory mechanisms", "participation and voice" and "governance in competency development in higher education for public health".

Finally, what does this mean for actors? Developing effective HRH plans clearly needs more than the technical capacities and tools. Acknowledging and dealing with governance should be part and parcel of the planning and implementation process as well. So far, little has been shared on governance and HRH and many questions remain unanswered. We hope that this thematic series stimulates readers to come to the fore with their experiences and document these. It is by mutual learning that we will learn how to better deal with governance.

## Competing interests

The authors declare that they have no competing interests.

## Authors' contributions

MD drafted the manuscript and TH extensively reviewed and contributed to the text. Both authors read and approved the final manuscript.
